# Complications of ent foreign bodies: a retrospective study

**DOI:** 10.1016/S1808-8694(15)30744-8

**Published:** 2015-10-19

**Authors:** Ricardo Rodrigues Figueiredo, Andréia Aparecida de Azevedo, Arthur Octavio de Ávila Kós, Shiro Tomita

**Affiliations:** 1 Master's degree in Otorhinolaryngology, UFRJ, Assistant Professor and Head of the ENT Service, Valenca Medical School, RJ; 2 Physician, otorhinolaryngologist. OTOSUL, Sul-Fluminense Otorhinolaryngology; 3 Doctoral degree in Otorhinolaryngology, Professor Emeritus of Otolaryngology, Medical School, Rio de Janeiro Federal University, UFRJ; 4 Doctoral degree in Otorhinolaryngology, Full Professor and Head of the Otorhinolaryngology Service, Medical School, UFRJ

**Keywords:** complications, foreign bodies, otolaryngology

## Abstract

Foreign bodies are one of the most common ENT (Ear, Nose and Throat) urgencies. Serious complications may occur, like tympanic perforations and bronchoaspiration, but they are uncommon.

**Aim:**

To analyze a 1356 foreign body series and establish causes for the complications, looking at prevention.

**Materials and methods:**

1356 patients with ear, nose and throat foreign bodies from the ENT Department of Souza Aguiar Hospital, in Rio de Janeiro, between 1992 and 2000, were analyzed in a retrospective study for parameters like age, gender, type and localization of the foreign body, time span between introduction and removal of the foreign body and complications.

**Results:**

The most common foreign bodies were beans and the most frequent age was between 1 and 4 years old. Ear foreign bodies were the most common, followed by nasal foreign bodies. Complications were statistically related to time, child's age and practical experience of the physician.

**Conclusion:**

Most of the situations related to ENT foreign bodies are avoidable. Improvements in Public Health Assistance and otolaryngologists' training are essential to avoid serious complications.

## INTRODUCTION

According to the literature,^1^, ^2^, ^3^, ^4^ foreign bodies are responsible, on average, for 11% of otorhinolaryngological emergencies; complications ensue in 22% of cases. Most of these complications are easily resolved, but occasionally severe conditions may emerge, such as tympanic perforation and bronchoaspiration.^4^^,^^5^

Decisive factors contributing to complications^3^^,^^4^ are:

removal attempts by laypersons and untrained health professionals;

• lack of medical experience in managing foreign bodies;
  lack of adequate hospital infrastructure;
  poor structure of the public health sector for dealing with otorhinolaryngological emergencies;
• foreign body remaining within the site for a long period, frequently due to the previous item.

Our purposes in this paper were:

to describe our 8-year experience in managing foreign bodies of the ear, nose, pharynx and larynx;

to analyze the main factors leading to complications by studying 1,356 foreign body removal cases and seeking forms of avoiding those complications.

## PATIENTS, MATERIAL AND METHOD

We conducted a retrospective analysis of 1,356 foreign body cases seen at our Unit between December 1992 and December 2000. Six parameters were taken into account: age, sex, site of foreign body (FB), type of FB, complications, duration of the period between FB introduction and removal and date of removal (between 1992 and 2000). About this last parameter, its aim was to establish a relation between the otorhinolaryngologist's experience and the presence of complications, given that the same medical doctor (the author) removed all FB. We also analyzed the relation between complications and other parameters; complications were classified as iatrogenic and non-iatrogenic (the set of complications was named “complications in general”). The chi-square (χ2) test and Fisher's exact test were used for statistical analyzing the relation between complications on the one hand and sex, age, type of FB, site, duration until removal and date of FB removal on the other. The significance level was 5%, that is, there was no statistical significance when the statistical test p value was equal to or below 0.05.

The surgical materials used for FB removal included nasal and auricular speculae, Bruennings tongue retractors, rigid 4 mm diameter optic fibers (70°, 0° and 30°), Kelly and bayonet forceps, an optic fiber laryngoscope, Hartmann and alligator forceps, blunt and pointed hooks, syringes for ear lavage and an electrical ear cleaner. The Research Ethics Committee of the Souza Aguiar Municipal Hospital approved this study (protocol number 32/2003).

## RESULTS

OF 1,356 FB cases, 753 (55.53%) were in the ear, 420 (30.97%) were nasal, 179 (13.21%) were pharyngeal, and 4 (0.29%) were laryngeal FBs. There were 129 pharyngeal FBs lodged in the tonsils (72.06%), of which 65 were in the right tonsil and 64 in the left tonsil. There were 31 FB cases (17.32%) in the base of the tongue, 12 (6.70%) in the vallecula, 3 (1.68%) in the right supratonsillar recess (STR), 2 (1.12%) in the hypopharynx, and 2 (1.12%) in tonsillar areas (tonsillectomized patients), of which 1 was to the right and 1 to the left.

**Chart 1:**
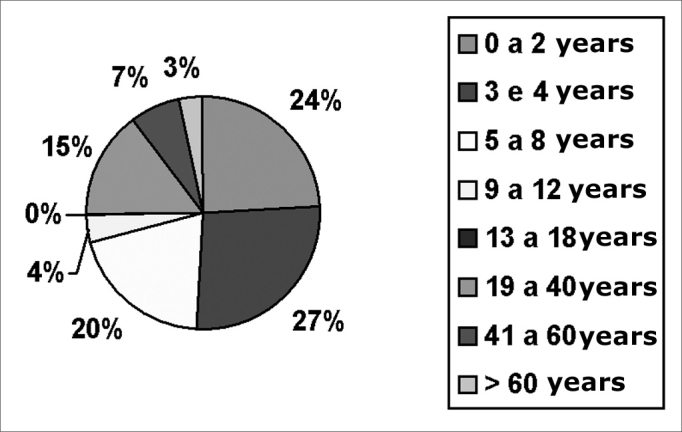
Distribution by age range

There were 674 cases (49.70%) in females and 682 cases (51.30%) in males. The age distribution may be seen in Chart 1.

**Chart 2:**
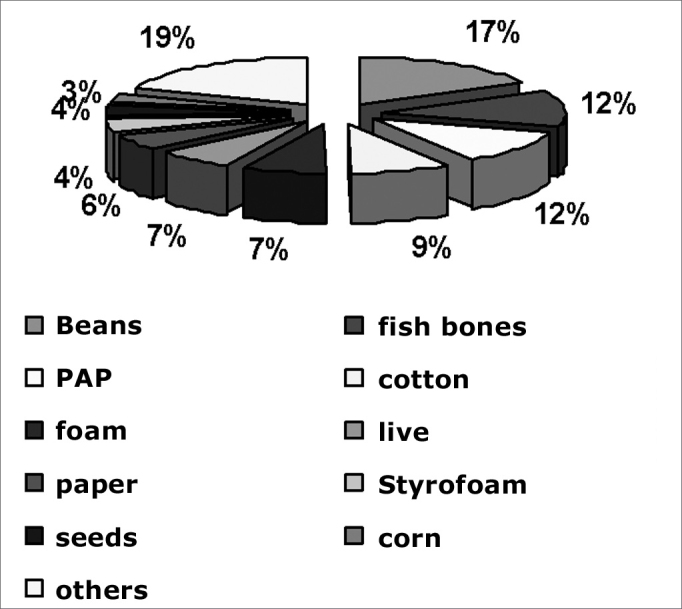
FB types

The type of FBs may be seen in Chart 2. The acronym SPA means “Small Plastic Artifacts.” Beans and corn grains were not included in this chart and were analyzed separately.

**Chart 3:**
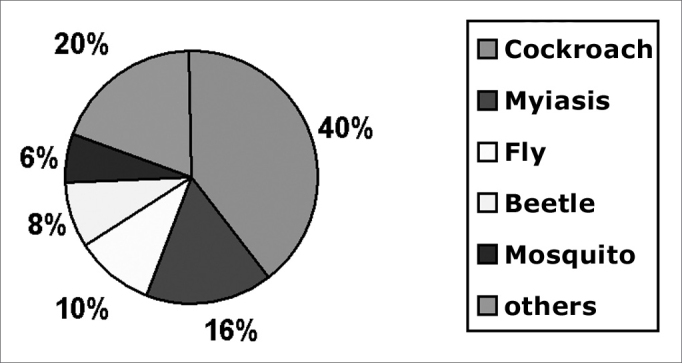
Live FB

Live FBs are described in Chart 3.

**Chart 4:**
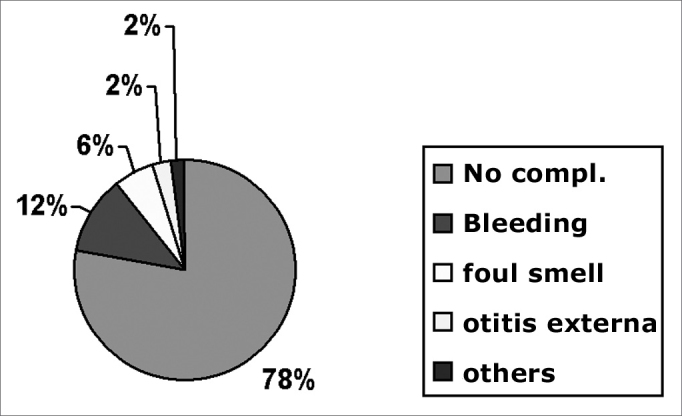
Complications

There were no complications in 1,055 cases (77.80%). Complications were seen in 301 cases (22.20%), including those resulting from removal procedures. Complications were iatrogenic in 159 cases (11.70%). The types of complications may be seen in Chart 4.

**Chart 5:**
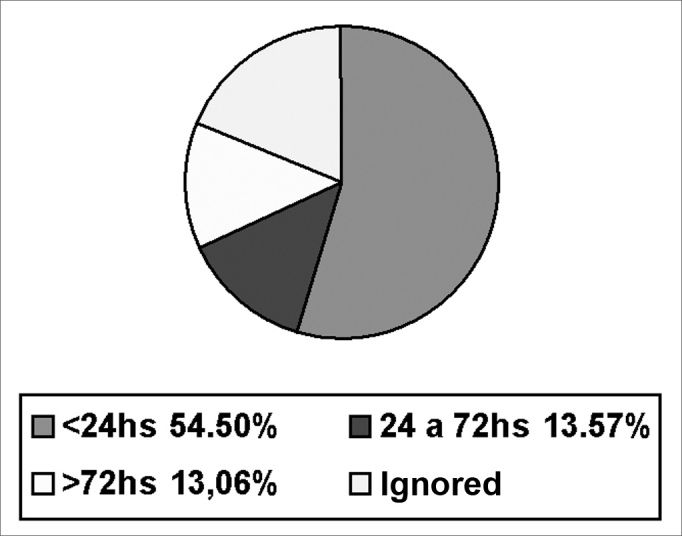
Time span between FB introduction and removal

Chart 5 shows the time elapsed between FB introduction and removal. The mean duration was 9.71 hours.

Table 1 shows the frequency (n) and the percentage (%) for sex, age, type, site, duration and date related to the presence and absence of complications in general. The chi-square (χ2) test was used for the statistical analysis. After this point, bean and corn grains were included in the item “seeds”.

**Table 1:** Statistical analysis for complications in general.

Code	Variables	category	with complications		no complications		p value
			n	%	n	%	
x2	sex	male	518	49.1	164	54.5	0.099
	(n = 1356)	Female	537	50.9	137	45.5	
		<= 2 years	99	32.9	212	20.1	
		3 a 6	126	41.9	377	35.7	
x3_a	age (n = 1356)	7 a 10	36	12.0	95	9.0	< 0.0001
		11 a 18	9	3.0	78	7.4	
		19 a 50	27	9.0	216	20.5	
		> 50	4	1.3	77	7.3	
		Live	22	35.5	74	21.2	
x4_	Type	Fish spine	6	9.7	159	45.6	< 0.0001
	(n = 411)	SPA	22	35.5	76	21.8	
		Seeds	12	19.4	40	11.5	
		ear	157	52.3	596	56.7	
x6_a	Site (n = 1352)	Nose	137	45.7	283	26.9	< 0.0001
		Pharynx	6	2.0	173	16.4	
x8_a	Duration (n = 1103)	<= 24 h	109	52.9	709	79.0	< 0.0001
		24 a 72 h	35	17.0	106	11.8	
		> 72 h	62	30.1	82	9.1	
		92 a 94	103	34.2	349	33.1	
x9	Date (n = 1356)	94 a 96	63	20.9	323	30.6	0.004
		96 a 98	88	29.2	266	25.2	
		98 a 00	47	15.6	117	11.1	

A significant association was found between:
• child age and presence of complications in general (p = 0.0001). • the type of FB (live and SPA) and presence of complications in general (p = 0,0001). • the site of the FB (nose) and presence of complications in general (p < 0.0001). • the time until removal and presence of complications in general (p < 0.0001). This means that there was a significant relation between complications in general and duration > 24 hours. On the other hand, there was also a time < 24 hours relation in the group with no complications. • the date and presence of complications in general (p = 0.004). This means that there was a significant relation between complications in general and time from 1996 to 2000. No significant association was found between sex and complications in general (p=0.099).

Table 2 shows the frequency (n) and the percentage (%) for sex, age, type, site, duration and date related to the presence and absence of iatrogenic complications. The chi-square (χ2) test was used for the statistical analysis.

**Table 2:** Statistical analysis of iatrogenic complications.

Cod.	Variables	category	Iatrogenic complication		Non-iatrogenic complication or no complication		p value
			n	%	n	%	
x2	Sex (n = 1356)	Male	89	56.0	593	49.5	0.12
		Female	70	44.0	604	50.5	
		<= 2 years	41	25.8	270	22.6	
		3 a 6	83	52.2	420	35.1	
x3_a	Age (n = 1356)	7 a 10	21	13.2	110	9.2	< 0.0001
		11 a 18	3	1.9	84	7.0	
		19 a 50	9	5.7	234	19.6	
		> 50	2	1.3	79	6.6	
		Live	5	11.9	91	24.7	
x4_	Type (n = 411)	Fish spine	6	14.3	159	43.1	< 0.0001
		SPA	21	50.0	77	20.9	
		Seeds	10	23.8	42	11.4	
		ear	98	62.0	655	54.9	
x6_a	Site (n = 1352)	Nose	54	34.2	366	30.7	0.001
		Pharynx	6	3.8	173	14.5	
x8_a	Duration (n = 1103)	<= 24 h	100	80.7	718	73.3	0.20
		24 a 72 h	11	8.9	130	13.3	
		> 72 h	13	10.5	131	13.4	
		92 a 94	68	42.8	384	32.1	
x9	Date (n = 1356)	94 a 96	27	17.0	359	30.0	< 0.0001
		96 a 98	35	22.0	319	26.7	
		98 a 00	29	18.2	135	11.3	

A significant association was found between:
• child age and presence of iatrogenic complications (p = 0.0001). • the type (SPA and seeds) and presence of iatrogenic complications (p = 0.0001). • the site (ear) and presence of iatrogenic complications (p = 0.001). • the date and presence of iatrogenic complications (p = 0.0001). This means that there was a significant relation between iatrogenic complications and the date from 1992 to 1992–1994. • There was no significant association between sex (p = 0.12) and duration (p = 0.20), and presence of iatrogenic complications.

These results up to this point showed that the non-iatrogenic complications groups had particular features. The three groups, therefore, were analyzed separately to improve characterization according to the variables we investigated.

Table 3 shows the frequency (n) and the percentage (%) for sex, age, type, site, duration and date related to the presence and absence of iatrogenic and non-iatrogenic complications, and no complications. The chi-square (χ2) test and Fisher's exact test was used for the statistical analysis.

**Table 3:** Statistical analysis of complications (iatrogenic, non-iatrogenic) and no complications.

Cód.	Variables	category	iatrogenic compl		non-iatrogenic compl		no complication		p value
			n	%	n	%	n	%	
x2	Sex (n = 1356)	male	89	56,0	75	52,8	518	49,1	0,22
		female	70	44,0	67	47,2	537	50,9	
		<= 2 years	41	25,8	58	40,9	212	20,1	
		3 a 6	83	52,2	43	30,3	377	35,7	
x3_a	Age (n = 1356)	7 a 10	21	13,2	15	10,6	95	9,0	< 0,0001
		11 a 18	3	1,9	6	4,2	78	7,4	
		19 a 50	9	5,7	18	12,7	216	20,5	
		> 50	2	1,3	2	1,4	77	7,3	
		Live	5	11,9	17	85,0	74	21,2	
x4_	Type (n = 411)	Fish spine	6	14,3	0	0,0	159	45,6	< 0,0001
		SPA	21	50,0	1	5,0	76	21,8	
		Seeds	10	23,8	2	10,0	40	11,5	
		ear	98	62,0	59	41,6	596	56,7	
x6_a	Site (n = 1352)	Nose	54	34,2	83	58,5	283	26,9	< 0,0001
		Pharynx	6	3,8	0	0,0	173	16,4	
		<= 24 h	100	80,7	9	11,0	709	79,0	
x8_a	Duration (n = 1103)	24 a 72 h	11	8,9	24	29,3	106	11,8	< 0,0001
		> 72 h	13	10,5	49	59,8	82	9,1	
		92 a 94	68	42,8	35	24,7	349	33,1	
x9	Date (n = 1356)	94 a 96	27	17,0	36	25,4	323	30,6	< 0,0001
		96 a 98	35	22,0	53	37,3	266	25,2	
		98 a 00	29	18,2	18	12,7	117	11,1	

A significant association was found between:
• child age and presence of complications (p < 0.0001). This means that there was a significant relation between age (until age 10 years) and complications (iatrogenic and non-iatrogenic). On the other hand, the group with no complications was related with adults. • the type and presence of complications (p < 0.0001). This means that there was a significant relation between iatrogenic complications and SPA and seeds, and that there was a significant relation between non-iatrogenic complications and insects. On the other hand, the group with no complications was related with fish spine. • the site and presence of complications (p < 0.0001). This means that there was a significant relation between iatrogenic complications and the ear, and that there was a significant relation between non-iatrogenic complications and nose. On the other hand, the group with no complications was related with the pharynx. • the duration and presence of complications (p < 0.0001). This means that there was a significant relation between non-iatrogenic complications and duration > 24 hours. On the other hand, the groups with no complications and with iatrogenic complications were related with duration < 24 hours. • the date and presence of complications (p < 0.0001). This means that there was a significant relation between iatrogenic complications and the date (1992–1994), and that there was a significant relation between non-iatrogenic complications and the date (1996–1998). On the other hand, the group with no complications was related with the date (1994–1996).

There was no significant association (p = 0.22) between sex and the type of complication.

## DISCUSSION

The study of FBs is fascinating, presenting many regional peculiarities. Otorhinolaryngology deals with most of the natural orifices that are habitually exposed, such as the mouth, nostrils and ears.^4^ The esophagus and lower airways are affected indirectly, as FBs must first pass through the pharynx or the nasal fossae. Oropharyngeal and nasal fossae FBs are potentially esophageal and bronchial FBs.^3^, ^4^, ^5^

FBs in the ear may lead to tympanic perforation and deafness, particularly if there is secondary infection. Severe nose bleeding associated with FBs is not uncommon; fish spine may lead to peritonsillar abscesses.^4^^,^^6^^,^^7^

Our findings about the site of FBs are in agreement with the literature,^8^, ^9^, ^10^, ^11^, ^12^ in which ear FBs predominated, followed by those in the nose and throat. In our opinion, nose and throat FBs are more easily eliminated by physiological mechanisms, such as sneezing, coughing and the nausea reflex. The natural projection of tonsils into the oral cavity explains its position as the most common site for impacted pharyngeal FBs. There were no statistically significant differences in the sex distribution, which is in agreement with the literature.^2^, ^3^, ^4^^,^^11^^,^^13^

**Figure 1:**
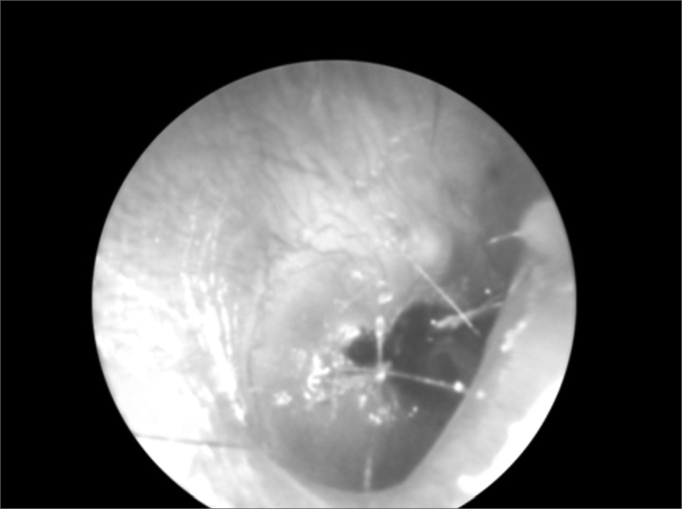
Tympanic perforation by a foreign body (bamboo fragment).

**Figure 2:**
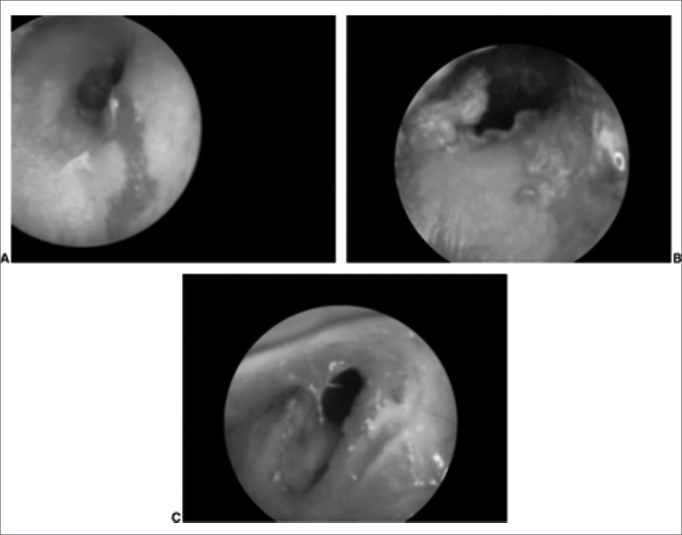
External auditory canal skin laceration.

**Figure 3:**
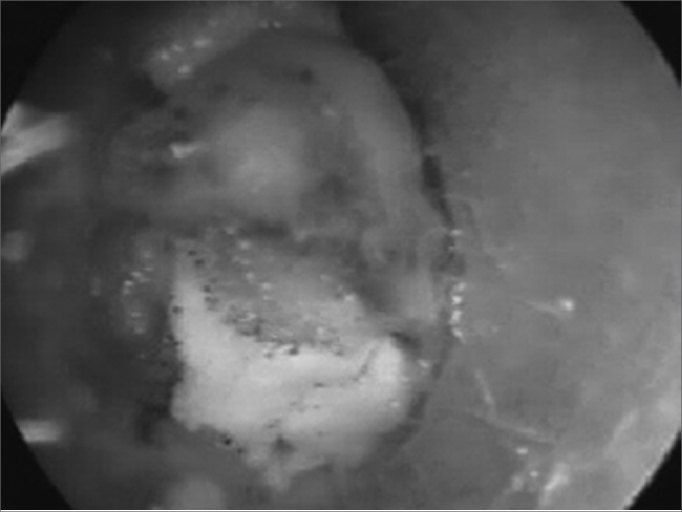
Foreign body associated otomycosis (cotton fragment).

**Figure 4:**
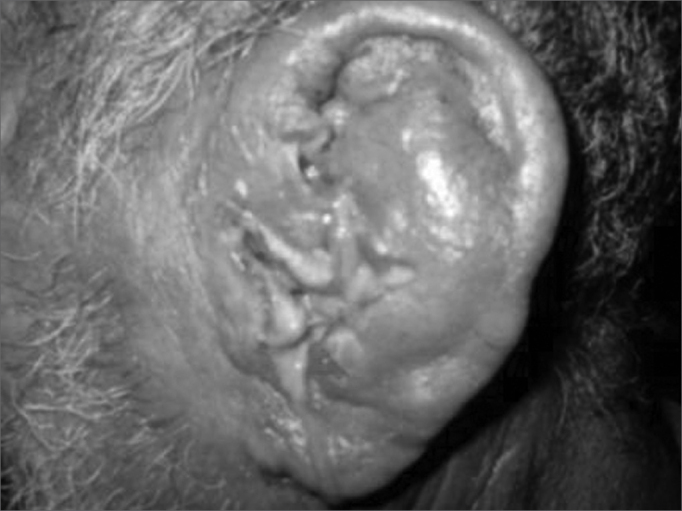
Perichondritis secondary to myiasis.

The age distribution showed a clear predominance of children aged between 1 and 4 years (47.64% of cases). Nasal FBs are almost exclusively found in children; pharyngeal FBs are more commonly found in adults; the distribution of ear FBs is more balanced, with a slight predominance in children. These findings are also in agreement with the literature^8^^,^^9^^,^^13^

Beans were the most common FB (17.18%), the most frequent FB in the ear (23.11%) and the third most frequent FB in the nose (14.76%). A high rate of fish spine as pharyngeal FBs usually reflects lack of care in preparing meals, specifically small fish, which are usually the least expensive. SPA are parts of toys and various other objects, such as buttons and food package seals. Toys and other objects bought from street vendors are especially dangerous; most of them contain no recommendations about age. Cotton fragments reflect popular cleaning habits and methods for relieving otological pruritus. Foam fragments, usually from ruptured pillows and mattresses, were the most common nasal FBs found.

Live FBs require specific comments. At our unit, the most common live FBs were cockroaches, followed by myiasis, mosquitoes and beetles.^4^^,^^15^ Cockroaches generally enter the external auditory canal from the ground, particularly in people who habitually (or for lack of choice) sleep on the floor.^15^^,^^16^ The clinical picture is dramatic and painful; the recommended approach is to first kill the insect by instilling oily substances, alcohol or ether into the external auditory canal. Live FBs are generally related to poor hygiene.^4^^,^^15^ The most frequently found fly associated with myiasis in Brazil is the Cochliomya hominivorax, found in ear and nasal fossae infestations.^15^^,^^16^ Complications of myiasis in the nose are common, such as septal perforation, necrosis of the turbinates and orbitary complications. Larvae should be surgically removed as much as possible and parenteral antibiotics (clyndamicin or crystalline penicillin associated with ceftriaxone) should be given.^4^^,^^15^ Some studies have suggested using ivermectine.^17^

Complications were found in 22.20% of cases. Marques et al. noted a higher rate of complications in cases that had been previously manipulated by non-otorhinolaryngologists, other health professionals, the patients themselves and other laypersons.^8^^,^^9^^,^^14^

In our data, the most frequent complications were bleeding (51.83% of complications), fetidness (28.57%) and external otitis (10.30%). There were fewer serious complications such as necrosis (1.33%) and tympanic perforation (0.99%). Bleeding usually is mild; in our series there was no need for contention measures. Fetidness occurs due to secondary bacterial infection, more commonly in nasal FBs, especially with hygroscopic materials such as foam fragments, paper and cotton.^2^, ^3^, ^4^ Some types of complications are shown below:

There was no statistically significant difference between sexes relative to complications. The statistically significant difference between complications and age below 10 years is probably due to agitation of the child during removal. A relation between the presence of iatrogenic complications and certain types of FBs (such as SPA and seeds) has led us to conclude that these FBs are technically more difficult to remove. Non-iatrogenic complications showed a statistically significant with live FBs, possibly due to an association with inflammation and infection.

Our data reveal a statistically significant relation between non-iatrogenic complications and nasal FBs, probably due to a higher incidence of secondary infection, which results in fetidness. Ear FBs were associated with iatrogenic complications, probably due to the tortuous anatomy of the external auditory canal, which makes removal more troublesome.

On the time period between FB insertion and removal, there was a statistically significant relation between non-iatrogenic complications and longer permanence FBs (over 72 hours); we conclude that delays in removing FBs increase the frequency of non-iatrogenic complications, which reinforces the importance of prompt treatment. There was a statistically significant relation between iatrogenic complications and FBs in place for less than 24 hours. We believe that this occurs because patients are more agitated soon after FB insertion. The incidence of FBs removed with no complications is also related to early treatment.

Finally, there was a higher incidence of iatrogenic complications in the first two years of our series, from 1992 to 1994. These findings indicate that professional experience in managing FBs is important; two reasons are given: improved manual dexterity with experience, and better decision-making about using sedation or general anesthesia. These findings reinforce the need for higher-quality teaching about urgencies in graduation courses.

Data on the time between FB insertion and removal show that removal takes place within the first 24 hours in 54.50% of cases, between 24 and 48 hours in 13.57% of cases, and after 72 hours in 13.06% of cases. This time period could not be established in 18.95% of cases, particularly in children that are wary of explaining their acts to caretakers.

## CONCLUSION

Our data describes the series in one of the major South American ENT urgency service. Findings in general are in agreement with the literature; there are local peculiarities, such as a predominance of beans as the most frequent FB. Iatrogenic complications were related to ear FBs, children, SPA, seeds, less than 24 hours elapsed between FB insertion and removal, and lack of professional experience in managing FBs. Non-iatrogenic complications were related with live FBs and long duration.

Based on the abovementioned conclusions, certain measures are suggested that might avoid complications, as follows:

1) Informing patients to immediately seek an otorhinolaryngologist in FB cases, especially for the treatment of live FBs.
2) Increased care by otorhinolaryngologists with technically difficult to remove FBs, such as seeds and SPA, especially in ears and in children, in whom removal under sedation or general anesthesia should be considered.
3) Improved teaching about urgencies in ENT graduation courses.
